# Can Novel Treatment of Age-Related Macular Degeneration Be Developed by Better Understanding of Sorsby’s Fundus Dystrophy

**DOI:** 10.3390/jcm4050874

**Published:** 2015-05-04

**Authors:** Hanae C. Y. Gourier, N. Victor Chong

**Affiliations:** Oxford Eye Hospital, Oxford University Hospitals, Oxford, OX3 9DU, UK; E-Mail: hanae.gourier@gmail.com

**Keywords:** Sorsby’s fundus dystrophy, age-related macular degeneration, Bruch’s membrane, TIMP-3, choroidal neovascularisation, geographic atrophy

## Abstract

Sorsby’s Fundus Dystrophy (SFD) is a rare autosomal dominant maculopathy that shares many clinical features with Age-Related Macular Degeneration (AMD). It is caused by a mutation in a single gene, TIMP-3, which accumulates in Bruch’s membrane (BM). BM thickening and TIMP-3 accumulation can also be found in AMD. From our understanding of the pathophysiology of SFD we hypothesize that BM thickening could be responsible for making the elastic layer vulnerable to invasion by choriocapillaris, thereby leading to choroidal neovascularization in some cases of AMD, whilst in others it could deprive the retinal pigment epithelium of its blood supply, thereby causing geographic atrophy.

## 1. Introduction

Sorsby’s Fundus Dystrophy (SFD) is a rare autosomal dominant maculopathy. Sorsby first described it in 1949, when he identified five families whose members developed central visual loss before their forties, and whose fundal appearances resemble that of patients suffering with age-related macular degeneration (AMD) [1].

**Figure 1:**
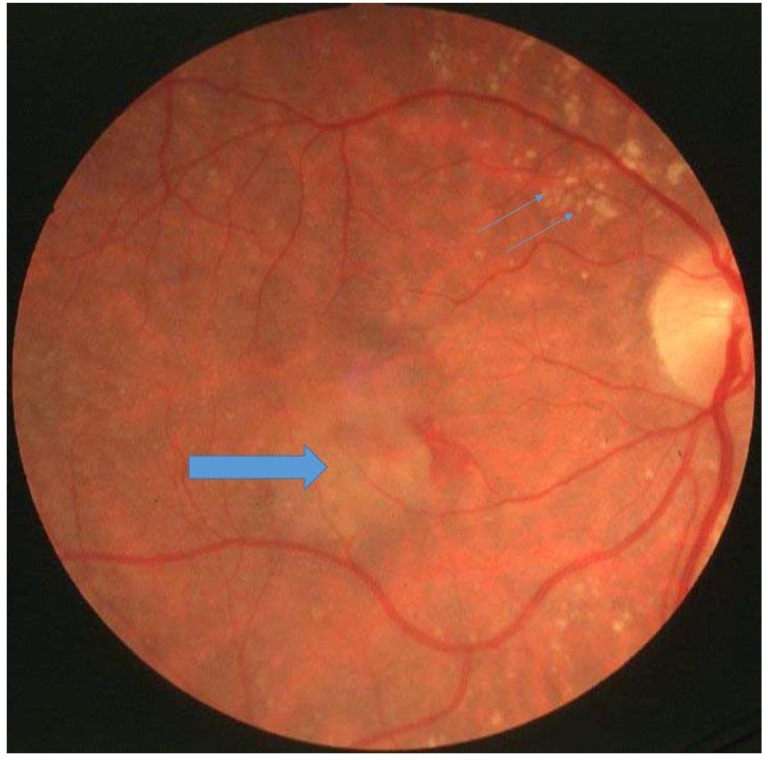
Patient with Sorsby’s fundus dystrophy with drusen (small arrows) and choroidal neovascularisation (large arrow).

**Figure 2:**
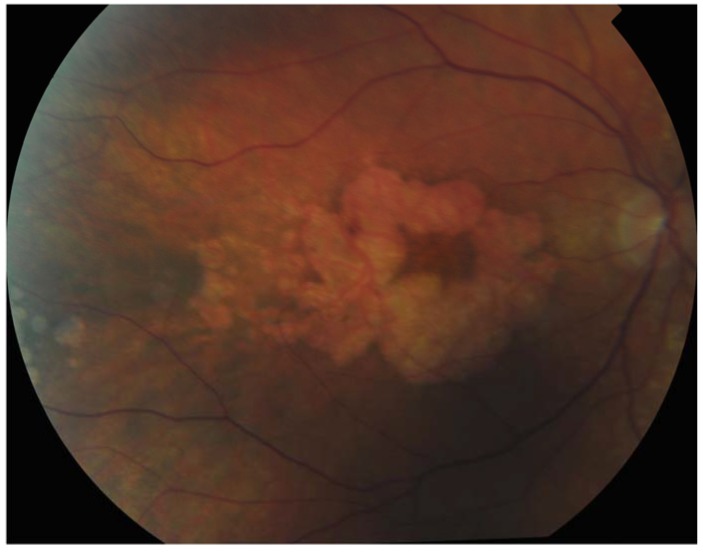
Patient with Sorbsy’s fundus dystrophy showing geographic atrophy with foveal sparing.

Similar to AMD, an early hallmark of SFD is the presence of yellow drusen-like deposits in the macula. These yellow deposits are in the same anatomical location of drusen in the sub-retinal pigment epithelial (RPE) space but the material is different from drusen. Again, like AMD, SFD can progress in one of two ways: some patients develop choroidal neovascularisation (CNV) (Figure 1) and suffer from acute visual loss when these new vessels start to leak, whilst others undergo progressive central visual loss as they develop pigment epithelial atrophy similar to geographic atrophy (GA) (Figure 2) in AMD [2,3,4]. Unlike AMD, one of the earliest symptoms of SFD is nyctalopia and in some cases significantly restricted peripheral vision.

While SFD is remarkably rare, AMD has important public health implications, being the most common cause of blindness in the elderly in developed countries. AMD has a complex aetiology that involves multiple alleles and we have yet to fully understand it. Interestingly, a large-scale genome-wide association study (GWAS) that analysed CNV and GA cases separately has shown that the *ARMS2* risk alleles preferentially associate with CNV whereas the *CFH* risk alleles preferentially associate with GA [5]. It is now also clear that complement pathways are implicated in the pathophysiology of AMD [6]. On the other hand, SFD can be explained by a series of mutations in a single gene that is now well described. Considering its straightforward genetics and the clinical and pathological similarities between the two conditions, SFD has always sparked a lot of interest as a model for AMD.

In this review, we propose a hypothesis on the development of AMD based on our observations of the pathophysiology underlying SFD. It could explain the phenotype in which both CNV and GA can be presented without there being complement pathways abnormalities. It may also be significant in the Asian population for whom the CFH risk allele has a low association [5]. Furthermore, understanding SFD might allow us to develop new therapies for AMD.

## 2. The Tissue Inhibitor of Metalloproteinases-3 Gene is Involved in SFD and in Some Cases of AMD

SFD is caused by mutations in the Tissue Inhibitor of Metalloproteinases-3 (TIMP-3) gene [7,8,9,10,11]. TIMP-3 is a member of the Tissue Inhibitors of Metalloproteinase family, which comprises TIMP-1, -2, -3, and -4 [12]. Their role is to inhibit matrix metalloproteinases (MMPs) that degrade the extracellular matrix (ECM). All members of the TIMP family are soluble proteins except for TIMP-3 [13]. TIMP-3 has the particularity of being expressed by RPE cells [14], before being incorporated and sequestered in the ECM where it acts to control ECM remodelling.

Considering the phenotypic similarities between SFD and AMD, TIMP-3 has long been a suspect in the pathophysiology of AMD. However, no link was found between the two until, in 2010, Chen *et al.* performed a GWAS in 2157 patients with AMD [15]. They identified a susceptibility locus for AMD in an intron of the synapsin III (SYNIII) gene, which also encodes TIMP-3. TIMP-3 is located approximately 100 kb upstream of this locus at rs9621532.

The association between rs9621532 and phenotype was later examined by Ardeljan *et al.* [16]. They stratified three large groups of patients with AMD by phenotype and studied the influence of various single nucleotide polymorphisms (SNPs) in the region of rs9621532. They identified an allele that had a moderate protective role for neovascular AMD in two of the study cohorts, as it was associated with a milder phenotype. The same group then quantified the influence of this allele on the expression of TIMP-3. They first demonstrated that the polymorphism was responsible for a reduction in the expression of TIMP-3 mRNA in cultured primary human fetal retinal pigment epithelium cells (hfRPE), after which they compared mRNA expression in ocular tissue of selected individuals with this protective allele, and discovered both TIMP-3 and SYN3 expression were reduced in homozygotes as compared with heterozygotes. Combining these results, they concluded that the mutated allele is protective of the more severe forms of AMD by reducing the expression of TIMP-3. These findings were unexpected. One would expect a reduction of TIMP-3 expression lead to less suppression of MMPs, in turn the MMPs would increase enzymatic activity leading to the destruction of the elastic layer in the Bruch’s membrane. However, it is possible that the increased MMP activities might allow waste material in the Bruch’s membrane to be cleared more quickly and, hence, reduce the Bruch’s membrane thickness, in turn leading to a protective effect.

## 3. Bruch’s Membrane Thickening Is a Key Pathological Feature of Both SFD and AMD

**Figure 3:**
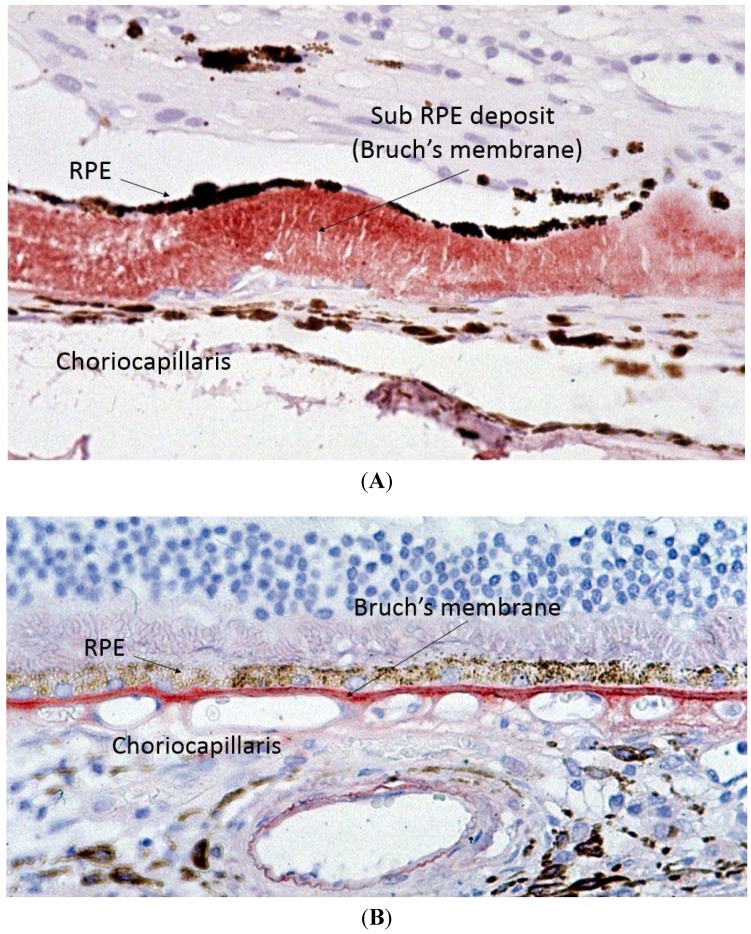
Light microscopy of Sorsby’s fundus dystrophy (**A**) showing extensive thickening of the Bruch’s membrane up to 30 microns as compared to normal (**B**) Bruch’s membrane thickness of one to two microns.

Capon *et al.* performed the first electron microscopy study on ocular tissue in a patient with SFD and described one of the key histopathological features of SFD, which is thickening of Bruch’s membrane (BM) (Figure 3) [2]. BM is a multi-layered membrane that separates the retinal pigment epithelium (RPE) and the choriocapillaris (CC). Its inner and outer layers are collagen-rich, whilst its core contains large amounts of elastin. The RPE gets its blood supply from the CC, and the BM is, therefore, responsible for the diffusion and transport of molecules from the CC to the RPE [17]. Whilst the initial description of SFD reported a maximal thickness of the deposit of up to 30 microns, more recent reports describe it can reach up to 60 microns [2,18]. It is easy to envisage how thickening of BM means it would act as a barrier to diffusion between the CC and RPE. Later studies demonstrated that the thickened BM stains very strongly for TIMP-3, which is therefore an integral part of this deposit [18,19]. Of note, once the RPE cells have died, the BM loses its TIMP-3 immunostaining ability [18].

The common mutations responsible for SFD are missense mutations that result in the addition of a cysteine residue, thereby encouraging the formation of aberrant intermolecular disulphide bridges [20]. Multimerisation does not affect the function of these mutants, which continue to behave like wild-type molecules functionally: they also sequester to the ECM and inhibit MMPs [21,22]. However, once multimerised, these mutants are much more resistant to degradation. These results confirm those of our previous findings, which demonstrated that the accumulation of TIMP-3 in BM is not due to increased TIMP-3 mRNA production, thereby suggesting it must be due to decreased degradation [23]. Further work has shown that dimerised TIMP-3 interacts with collagen VI molecules [24]. This interaction may also explain why both proteins are protected from normal metabolic processes. Patches and clusters of these aggregates can be observed in the thickened BM under electron microscopy (EM). Again, a parallel can be drawn with AMD, which shows similar molecular assemblies under EM.

The incidence of AMD increases drastically with age, and a number of changes occur in the BM as part of the normal ageing process, including thickening. These BM deposits accumulate preferentially in the macula, as do lesions in AMD [25]. Interestingly, this thickening also stains increasingly positive for TIMP-3 with increasing age in normal eyes [26,27]. However, TIMP-3 staining is at its strongest in the thickened BM of individuals affected with AMD.

The pathological features in SFD and AMD are similar, and since SFD is an autosomal dominant condition with full penetrance, it may be safe to assume that complement activation is not needed to develop this phenotype as not all SFD patients would also carry the high-risk CFH alleles. Nonetheless, it is possible that the CFH genotype might explain the phenotype variation of SFD. It does however remain unclear so as why SFD presents at a younger age and why some patients develop CNV whilst other develop GA in both SFD and AMD.

## 4. From TIMP-3, BM Thickening and Damage to the Elastic Layer to CNV (Neovascular AMD)

TIMP-3 is not only a matrix metalloproteinase (MMP) inhibitor; it is also capable of inhibiting angiogenesis by binding to VEGF-receptor 2 (VEGFR-2) [28]. In animal models, TIMP-3 deficiency results in increased susceptibility to choroidal neovascularisation (CNV) [29,30]. In SFD, the mutant protein is accumulating in BM and remains bioactive, meaning angiogenesis should be inhibited even further, and it should be protective in the development of CNV. This is an interesting property considering the aberrant growth of choroidal vessels in the later stages of both SFD and AMD and the fact that some patients with neovascular SFD are now being successfully treated with VEGF-inhibitors [31]. Furthermore, in both SFD and AMD, the genetic defects affect all RPE cells, and BM thickening is present over the entire retina and not just over the macula, which is the area that is predominantly affected [19]. It is therefore logical to suggest that increased BM thickness on its own is not enough to cause CNV.

This paradox can be explained by the increased turnover of the ECM of BM, which leads to weakening of the elastic layer. As we have previously described, the elastic layer in the BM of SFD patients is abnormal [19], which is also the case in AMD patients: the elastic layer in BM is also significantly altered compared to age-matched controls [17].

Therefore, despite the anti-angiogenic properties of TIMP-3, which should be protective against CNV, weakening of the elastic layer could mean that there is CNV invasion of the retina. This finding is consistent with the clinical observation that CNV invasion is most common in the macular area in both SFD and AMD, which is the area that has the thinnest and most porous elastic layer in the eye [17]. Complement pathway abnormalities are unlikely to play a significant role in the evolution of CNV once it is formed, and it remains unclear how complement pathway abnormalities lead to CNV formation. The damage caused to the elastic layer and the degradation of elastin might be the fundamental step required for CNV invasion in the retina. We have previously shown that elastin degradation is increased in AMD patients [32]. The elastin-derived peptides and elastin fragments that result from elastin degradation can induce choroidal endothelial cell migration [33]. Hence, protecting the elastin layer in BM could be an important yet understudied area in the prevention of CNV.

## 5. From TIMP-3 and BM Thickening to GA (Atrophic AMD)

There is still no treatment and only a little information about the development of GA both in SFD and AMD. Uncovering the pathophysiology underlying these atrophic processes with a view to develop treatments is, therefore, a major target of future research in the field. Based on genetic data that the complement pathways play a significant role in the development of GA, it is easy to accept that increased activation of complement pathways can induce RPE and CC cell death. Nevertheless, GA is also common in SFD patients who should not have complement pathway abnormalities.

We hypothesize that the aforementioned thickening in BM is the basic pathology responsible for atrophy of the outer retinal layers in SFD and, in some cases, of AMD. In AMD, drusen and other sub-RPE material contribute to the thickness of the Bruch’s membrane. This increased thickness decreases vascular support to the RPE, which degenerates. A number of previous works in animal models have shown that destruction of the RPE leads to CC atrophy, and that regeneration of the RPE is accompanied by resurgence of the CC [34,35]. Under hypoxic conditions, VEGF is preferentially secreted on the basal aspect of the RPE, and it is mainly detected on the endothelium of the CC that faces the RPE, thereby suggesting that it is secreted by the RPE for the CC [36]. This was later confirmed by high-resolution analysis of the RPE and choroid vasculature of eyes affected with dry AMD in particular [37,38]. We further hypothesize that this is the start of a vicious circle in which CC dropout leads to a further reduction in blood supply and nutritional support to the RPE, thereby further contributing to RPE cell death. It is clearly important to separate the clinical phenotype of those with GA due to mainly complement activation and that due to mainly BM thickening as the treatment might be very different. Indeed, there is already data to suggest that anti-complement therapy for GA might only be effective in those with specific complement factor I polymorphisms.

Controlling cellular matrix turnover might be an important step in reducing BM thickness and GA, and supporting the CC might be similarly important. With the advancement of retinal imaging technology, we can image the thickness of Bruch’s membrane in humans with increased accuracy. This might allow us to separate GA patients with primary RPE cell loss from those with secondary RPE cell loss due to BM thickening.

## 6. Conclusions

In conclusion, SFD is a rare maculopathy that shares many clinical and pathological traits with AMD, including the possibility to progress to either neovascularisation or atrophy. While neovascularisation has been well characterised, and can now be managed with anti-VEGF therapy, the prevention of CNV remains an illusive goal. Protecting the elastic layer might be crucial.

Furthermore, there is still a crucial lack of information and treatment options for the atrophic forms of both conditions. As opposed to AMD, SFD is a single-gene disorder and has a clear aetiology. This makes the study of its pathophysiology much less challenging and provides us with a possible model to explain the development of atrophy in cases of AMD with GA without systemic complement activation.

We hypothesize that the atrophy process starts with thickening of BM. This is accelerated in SFD due to a mutation in TIMP-3, which is normally sequestered in the ECM but is protected from degradation in its mutant form. Thickening of the BM is also a pathological feature of AMD, and it seems that TIMP-3 is also part of this process but its role remains unclear. This thickened membrane then acts as a barrier to the diffusion and transport of nutrients and metabolites between the CC and the RPE, a key process in triggering RPE cell death. RPE cell death leads to CC dropout, which itself will lead to further RPE degradation. We hypothesize this vicious circle is responsible for atrophy in SFD and possibly even in AMD. Controlling ECM turnover and preventing BM thickening might be a critical part in preventing the progression of GA.
